# Incidence of cardiac sarcoidosis in those with extra-cardiac disease without known cardiac involvement - a CMR study

**DOI:** 10.1186/1532-429X-18-S1-P252

**Published:** 2016-01-27

**Authors:** Theodore M Murphy, Deirdre F Waterhouse, Stephanie I James, Cliona Kenny, Rory O'Hanlon

**Affiliations:** Cardiovascular Imaging Dept, Blackrock Clinic, Dublin, Ireland

## Background

Cardiac death is the leading cause of mortality in patients with sarcoidosis. However, the incidence of cardiac sarcoidosis in those with extracardiac disease has yet to be formally examined. Cardiovascular magnetic resonance (CMR) imaging with late gadolinium enhancement (LGE) may identify the myocardial infiltration of cardiac sarcoidosis (CS), and thus allow risk stratification of these at risk individuals. We therefore investigated the incidence of CS in patients with extracardiac sarcoidosis without cardiac manifestations using CMR-LGE.

## Methods

Seventy-two patients with clinically and radiologically diagnosed pulmonary sarcoidosis, without signs or symptoms of cardiovascular involvement, were enrolled over a three-year period. LGE-CMR was performed on all patients. The presence of LGE in the left ventricular myocardium was considered diagnostic for CS. Patients were classified as CS or non-CS based on the CMR-LGE findings.

## Results

Mean age was 51 ± 19 years, with a female predominance (61%). CS was detected in nineteen patients (23%). When compared to those patients without LGE, patients with CS had higher rates of previously documented non-sustained ventricular tachycardia (22% vs. 5%), a greater prevalence of an abnormal ECG (52% vs. 18%) and a higher heart rate during CMR (82bpm vs. 72bpm).

## Conclusions

CMR should be considered as an adjunct to conventional diagnostic workup in all patients with pulmonary sarcoidosis. Long-term follow-up of consecutive patients with isolated CS is needed to determine the natural history and to accurately define rates of ventricular arrhythmias.

